# Accuracy of death certifications of diabetes, dementia and cancer in Australia: a population-based cohort study

**DOI:** 10.1186/s12889-022-13304-8

**Published:** 2022-05-06

**Authors:** Zhiwei Xu, Richard Hockey, Paul McElwee, Michael Waller, Annette Dobson

**Affiliations:** 1 School of Public Health, Faculty of Medicine, University of Queensland, 288 Herston Road, Brisbane, QLD 4006 Australia; 2 University of Queensland, NHMRC Centre for Research Excellence On Women and Non-Communicable Diseases (CRE-WaND), Brisbane, QLD Australia

**Keywords:** Death certification, Diabetes, Dementia, Cancer, Multimorbidity

## Abstract

**Background:**

National mortality statistics are only based on the underlying cause of death, which may considerably underestimate the effects of some chronic conditions.

**Methods:**

The sensitivity, specificity, and positive and negative predictive values for diabetes (a common precursor to multimorbidity), dementia (a potential accelerant of death) and cancer (expected to be well-recorded) were calculated from death certificates for 9 056 women from the 1921–26 cohort of the Australian Longitudinal Study on Women’s Health. Log binomial regression models were fitted to examine factors associated with the sensitivity of death certificates with these conditions as underlying or contributing causes of death.

**Results:**

Among women who had a record of each of these conditions in their lifetime, the sensitivity was 12.3% (95% confidence interval, 11.0%, 13.7%), 25.2% (23.7%, 26.7%) and 57.7% (55.9%, 59.5%) for diabetes, dementia and cancer, respectively, as the underlying cause of death, and 40.9% (38.8%, 42.9%), 52.3% (50.6%, 54.0%) and 67.1% (65.4%, 68.7%), respectively, if contributing causes of death were also taken into account. In all cases specificity (> 97%) and positive predictive value (> 91%) were high, and negative predictive value ranged from 69.6% to 84.6%. Sensitivity varied with age (in different directions for different conditions) but not consistently with the other sociodemographic factors.

**Conclusions:**

Death rates associated with common conditions that occur in multimorbidity clusters in the elderly are underestimated in national mortality statistics, but would be improved if the multiple causes of death listed on a death certificate were taken into account in the statistics.

**Supplementary Information:**

The online version contains supplementary material available at 10.1186/s12889-022-13304-8.

## Background

National mortality statistics derived from death certificates are commonly used to measure the health of populations and inform public policy formulation [1]. The death certificate, formally known as the Medical Certificate of Causes of Death, has two parts: Part I “Disease or condition directly leading to death” and Part II “Other significant conditions contributing to the death, but not related to the disease or condition causing it” [2, 3]. Mortality rates are based on the underlying cause of death which is derived from the conditions recorded in Part I only. Consequently, underestimation of the mortality effects of some chronic conditions (e.g., diabetes [4, 5] and dementia [6–8]) has been a long-standing problem [9].

Data from well-designed longitudinal studies with information on participants’ health conditions are useful to ascertain the validity of causes listed on the death certificate and assess the value of including data from Part II. In the present study, we used data from the 1921–26 cohort of the Australian Longitudinal Study on Women’s Health (ALSWH) [10], a national population-based study with follow-up since 1996 and record linkage to multiple health data sources. We chose three conditions which might be expected to be recorded differently on the death certificate. These were: (i) diabetes (type 1 or type 2) because the onset of this condition is likely to occur years before death and be a precursor of multimorbidity, especially cardiovascular disease; (ii) dementia because it occurs at older age and may hasten deterioration and death; and (iii) cancer because this is well recorded in Australian Cancer Registries and so may be more likely to be mentioned on the death certificate. Diabetes and dementia are likely to cause or contribute to death and so could be expected to be recorded as the underlying cause of death or in Part II of the death certificate. However, successfully treated cancer would not necessarily be recorded if the death was from an unrelated cause.

The aims of the study were to: (1) quantify the accuracy (sensitivity, specificity, positive and negative predictive values) of death certification of diabetes, dementia and cancer as “the underlying cause of death” or as “the underlying or contributing cause of death”; (2) identify factors associated with the sensitivity of death certification of diabetes, dementia and cancer; and (3) identify the underlying cause of death recorded for people who had a record of diabetes, dementia or cancer in their lifetime but the condition was not the underlying cause on their death certificate.

## Material and methods

### Study design

ALSWH is an ongoing population-based cohort study of factors affecting the health and well-being of Australian women, and their use of health services. The details of ALSWH have been described elsewhere [10]. Women who were born in 1973–78, 1946–51 and 1921–26 were included in ALSWH initially, and in 2015, a new cohort of women who were born in 1989–95 was included in ALSWH [11].

The data used in this study were from the 1921–26 cohort. The women were randomly selected from the national universal health insurance database, with purposeful oversampling of those living in rural and remote areas. A total of 12 432 women responded to the first survey conducted in 1996, and these women were followed up every three years until 2011 and every six months since 2011. The survey data were linked to data from the universal subsidised health services: Medicare Benefits Schedule (MBS, mainly doctor visits), Pharmaceutical Benefits Scheme (PBS, medications), hospital admissions, cancer registration, aged care support, and the National Death Index. A total of 9 056 women who died between 1996 and 2017 were included in the present study, or for cancer analyses a total of 8 459 women who died between 1996 to 2016 (due to the availability of cancer registry data).

### Main outcome variables and covariates

Women who had a record of diabetes, dementia or cancer in their lifetime were identified through linkage of data from ALSWH surveys, MBS-subsidised services, PBS-subsidised prescriptions filled, hospital admissions, cancer registrations, and aged care assessments and services (see Tables S1.1, S1.2, S1.3 in Supplementary Material).

Causes of death were identified from linked data from the National Death Index which includes both the underlying cause of death and all conditions listed on the death certificate. Causes of death were coded using the International Classification of Diseases (ICD) versions 9 and 10 (see Table S2 in Supplementary Material). In Australia, these codes undergo automated processing which results in a set of causes identified as the underlying cause of death and contributing causes of death. In the present study the underlying cause of death is the one used in the national mortality statistics.

To examine sociodemographic factors associated with the sensitivity of death certifications of diabetes, dementia and cancer, we extracted information on the State/Territory, remoteness of the area of residence, education, country of birth, and marital status from the baseline ALSWH survey. State/Territory was included as there are some differences between jurisdictions in the death certification process. Area of residence was included as it could affect access to diagnostic and health services; it was categorized as metropolitan, regional area, and remote area. Education was included as a measure of socio-economic position and was categorized as low (no formal qualification), middle (school certificate or higher school certificate) and high (trade, apprentice, certificate, diploma, university degree, or higher degree). Country of birth was included as it could affect access to diagnostic and other services; it was categorized as Australia, other English-speaking countries, and other. Marital status was included as a marker of social support and source of proxy information; it was categorized as partnered (married or in a de facto relationship), or unpartnered (separated, divorced, widowed or never married). Year of death (< 2006, 2006–2011, and 2011–2017) was also included but needs to be interpreted carefully as it could mark changes in diagnostic or coding practices, but also it relates to age at death as all the women were born in the five-year period 1921–26 and death occurred between 1996 and 2017.

To examine the underlying causes of death recorded on the death certificates of those women who had diabetes, dementia, or cancer recorded in their lifetime but not on their death certificate, we categorized causes of death into 22 groups based on ICD codes (see Table S2 in Supplementary Material).

### Data analysis

Three analyses were conducted. We use diabetes as an example to clarify the details of each analysis. First, two-by-two tables were constructed with columns defined by whether or not a woman had a record of diabetes in her lifetime (this variable is treated as the ‘gold’ standard, or ‘truth’), and rows defined by whether or not diabetes was recorded on her death certificate, with separate tables for the underlying cause of death or an underlying or contributing cause of death. From these tables the following statistics (and 95% confidence intervals) were calculated: sensitivity, the proportion of women with the condition who had it reported on their death certificate, or true positives; specificity, the proportion of true negatives; positive and negative predictive values (which depend on the prevalence of the condition).

Second, log binomial regression models were fitted to quantify the association between the sociodemographic factors and the sensitivity of death certification of diabetes. The resulting rate ratios (RR) are sensitivity estimates relative to the reference category. These analyses were not undertaken for the other accuracy measures which were all much higher and less variable.

Third, for women with diabetes identified in their lifetime but without diabetes recorded as the underlying cause of death, we identified the five most commonly listed underlying causes of death.

These three analyses were repeated for dementia and cancer. Cancer registration data were used to identify women with cancer in their lifetime, but these data were only available up to 2016, so we only included women who died from 1996 to 2016 for the cancer analyses.

As women who had a record of diabetes or dementia in their lifetime were identified through multiple data sources, we conducted sensitivity analyses to calculate the sensitivity, specificity, and positive and negative predictive values of death certifications of diabetes and dementia by leaving one data source out each time.

All data analyses were conducted in SAS (version 9.4, SAS Institute Inc, Cary, NC, USA), and all figures were plotted using the ‘ggplot2’ package in R (version 3.5.3).

## Results

### Accuracy of death certifications of diabetes, dementia and cancer

The sensitivities, specificities, and positive and negative predictive values, with 95% confidence interval are shown in Table 1. Among the 9 056 decedents between 1996 and 2017, 2 164 women had diabetes in their lifetime, but only 12.3% had diabetes recorded as the underlying cause of death, and 40.9% had diabetes recorded as an underlying or contributing cause. In women with diabetes in their lifetime who died before 2006, 47.8% had diabetes recorded as an underlying or contributing cause, but this figure decreased to 43.0% in women who died during 2006–2011 and 35.7% in women who died during 2012–2017.

**Table 1:** Sensitivity, specificity, positive predictive value, and negative predictive value as percentages (with 95% confidence intervals) for diabetes, dementia, and cancer as the underlying cause of death, or underlying or contributing cause of death

	Sensitivity (%, number)	Specificity (%, number)	Positive predictive value (%, number)	Negative predictive value (%, number)
Underlying cause of death				
Diabetes	12.3 (11.0, 13.7), 267/2164	100.0 (99.9, 100.0), 6889/6892	98.9 (97.6, 100.0), 267/270	78.4 (77.6, 79.3), 6889/8786
Dementia	25.2 (23.7, 26.7), 835/3317	98.8 (98.5, 99.1), 5671/5739	92.5 (90.8, 94.2), 835/903	69.6 (68.6, 70.6), 5671/8153
Cancer	57.7 (55.9, 59.5), 1720/2980	99.1 (98.9, 99.4), 5430/5479	97.2 (96.5, 98.0), 1720/1769	81.2 (80.2, 82.1), 5430/6690
Underlying or contributing cause of death				
Diabetes	40.9 (38.8, 42.9), 884/2164	99.7 (99.5, 99.8), 6868/6892	97.4 (96.3, 98.4), 884/908	84.3 (83.5, 85.1), 6868/8148
Dementia	52.3 (50.6, 54.0), 1733/3317	97.1 (96.6, 97.5), 5571/5739	91.2 (89.9, 92.4), 1733/1901	77.9 (76.9, 78.8), 5571/7155
Cancer	67.1 (65.4, 68.7), 1998/2980	98.3 (98.0, 98.6), 5386/5479	95.6 (94.7, 96.4), 1998/2091	84.6 (83.7, 85.5), 5386/6368

There were 3 317 women who had dementia in their lifetime, and 25.2% of them had dementia recorded as the underlying cause of death, and 52.3% had dementia recorded as an underlying or contributing cause (Table 1). The sensitivity of death certification for dementia in women who died before 2006 (11.6% as underlying cause of death and 41.1% as an underlying or contributing cause) was lower than those who died during 2006–2011 (22.6% and 52.6%) or 2012–2017 (29.0% and 53.9%).

Among the 8 459 women who died between 1996 and 2016 there were 2 980 who had cancer in their lifetime, and 57.7% of them had cancer recorded as the underlying cause of death, and 67.1% had cancer recorded as an underlying or contributing cause (Table 1). The sensitivity of death certification for cancer in women who died before 2006 (72.9% as underlying cause of death and 81.8% as an underlying or contributing cause) was higher than it was in those who died during 2006–2011 (57.9% and 67.0%) and 2012–2016 (41.8% and 51.8%).

### Sociodemographic factors associated with the sensitivity of death certification

**Fig. 1:**
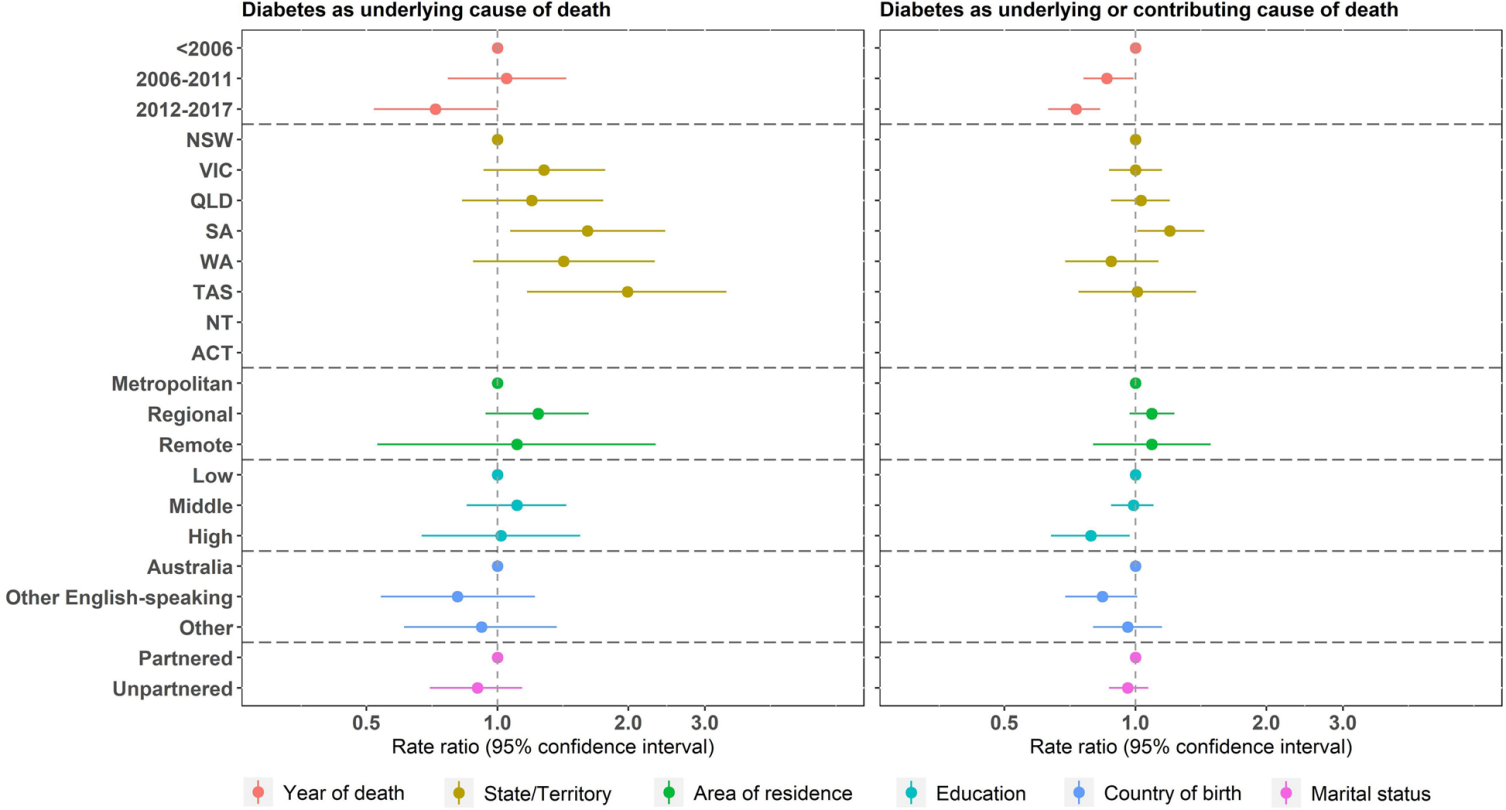
Association between sociodemographic factors and sensitivity of death certification in women with diabetes in their lifetime

For women with diabetes in their lifetime, those living in South Australia (adjusted rate ratio (RR): 1.61, 95% confidence interval (CI): 1.07, 2.43) or Tasmania (adjusted RR: 1.99, 95% CI: 1.17, 3.36) were more likely to have diabetes recorded as the underlying cause of death than those living in New South Wales (Fig. 1). Those who died during 2006–2011 (adjusted RR: 0.86, 95% CI: 0.76, 0.99) or 2012–2017 (adjusted RR: 0.73, 95% CI: 0.63, 0.83) were less likely to have diabetes recorded as an underlying or contributing cause, compared with those who died before 2006. Women with a higher level of education (adjusted RR: 0.79, 95% CI: 0.64, 0.97) were also less likely to have diabetes recorded as an underlying or contributing cause than women with less education.

**Fig. 2:**
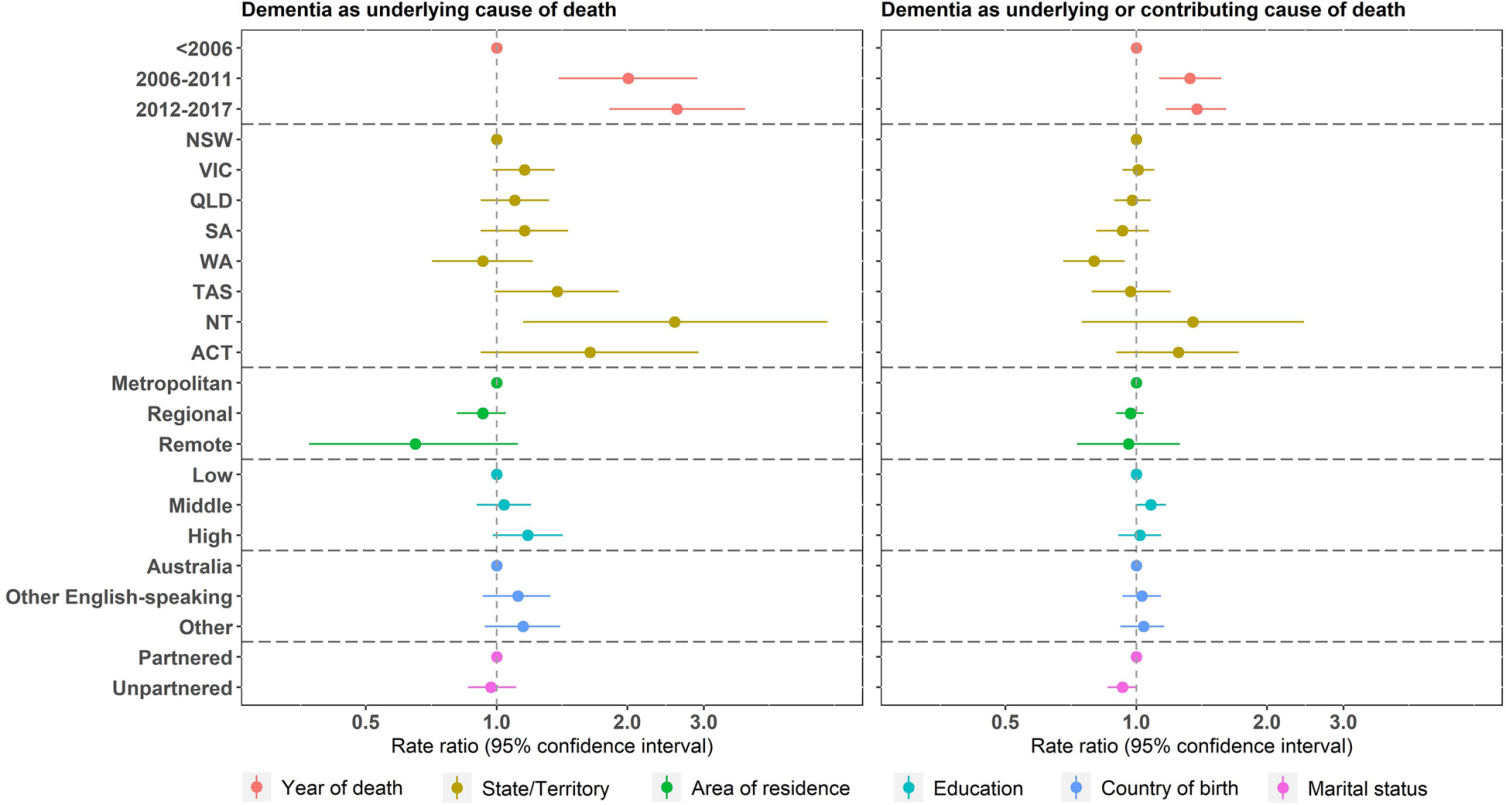
Association between sociodemographic factors and sensitivity of death certification in women with dementia in their lifetime

For women with dementia in their lifetime, those who died during 2006–2011 or 2012–2017 were more likely to have dementia recorded as the underlying cause of death (adjusted RR: 2.01, 95% CI: 1.39, 2.90 for 2006–2011; adjusted RR: 2.60, 95% CI: 1.82, 3.73 for 2012–2017) or as an underlying or contributing cause (adjusted RR: 1.33, 95% CI; 1.13, 1.57 for 2006–2011; adjusted RR: 1.38, 95% CI: 1.17, 1.61 for 2012–2017), compared with those who died before 2006 (Fig. 2). Unpartnered women were less likely to have dementia recorded as an underlying or contributing cause (adjusted RR: 0.93, 95% CI: 0.86, 1.00) than partnered women.

**Fig. 3:**
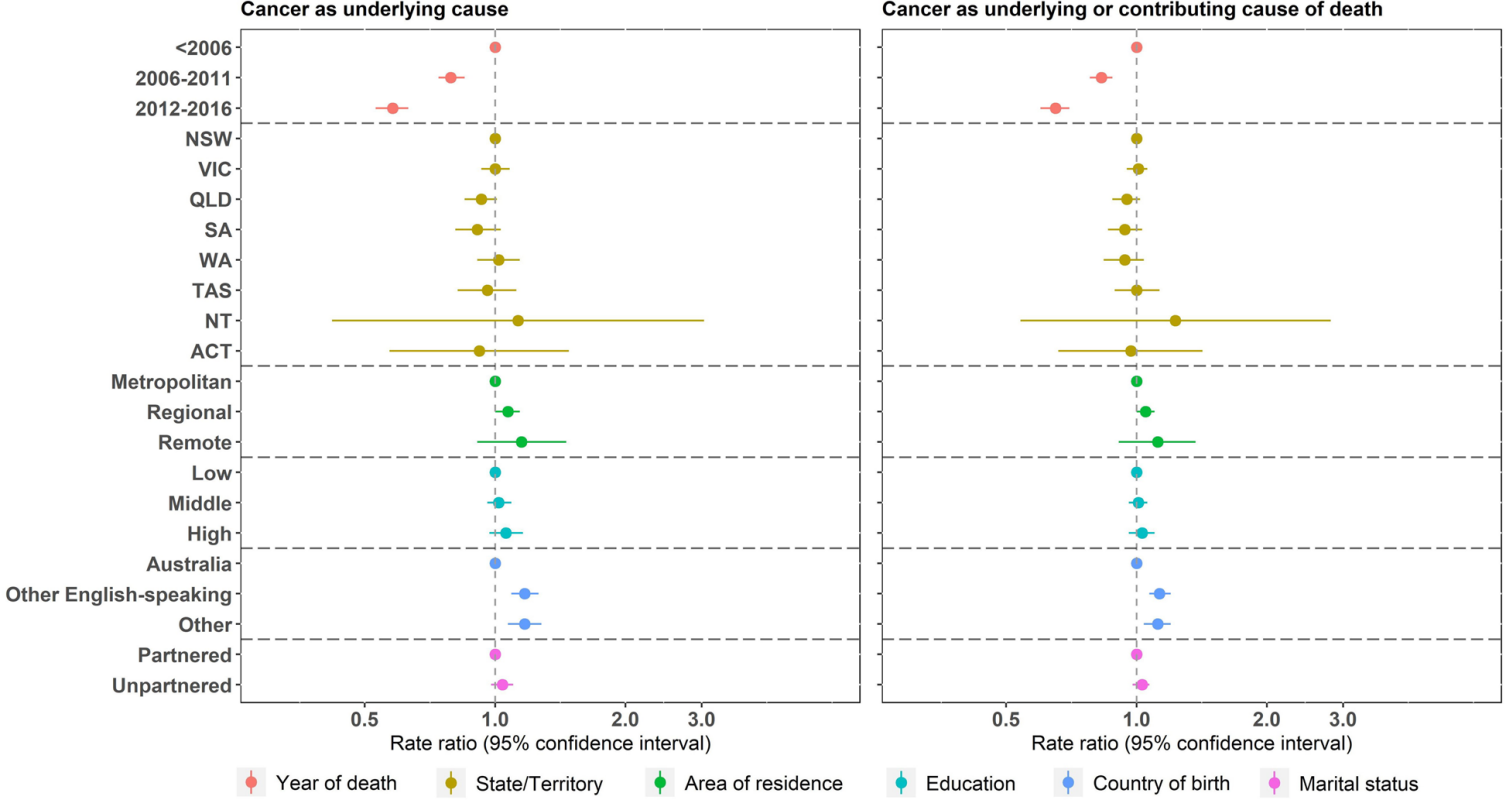
Association between sociodemographic factors and sensitivity of death certification in women with cancer in their lifetime

For women with cancer in their lifetime, those who died during 2006–2011 or 2012–2016 were less likely to have cancer recorded as the underlying cause of death (adjusted RR: 0.79, 95% CI: 0.74, 0.85 for 2006–2011; adjusted RR: 0.58, 95% CI: 0.53, 0.63 for 2012–2016) or as an underlying or contributing cause (adjusted RR: 0.83, 95% CI: 0.78, 0.88 for 2006–2011; adjusted RR: 0.65, 95% CI: 0.60, 0.70 for 2012–2016), compared with those who died before 2006 (Fig. 3). Women who were not born in Australia were more likely to have cancer recorded as the underlying cause of death, or as an underlying or contributing cause, compared with those who were born in Australia.

### For women with diabetes, dementia or cancer that was not recorded as their underlying cause of death, what was the underlying cause?

For women with diabetes, dementia or cancer but without the condition recorded as the underlying cause of death, the most likely underlying causes of death were similar: ischaemic heart disease, cerebrovascular disease, or other cardiovascular diseases (Table 2). For those with diabetes, dementia was the fourth most commonly recorded underlying cause. For those with dementia, diabetes was the fifth most commonly recorded underlying cause. For those with cancer, chronic lower respiratory disease was the fifth most commonly recorded underlying cause.

**Table 2:** The most common underlying causes of death in women with diabetes, dementia or cancers where these were not the underlying cause of death: ranking among underlying causes for this condition, number and percentage

Underlying cause	Conditions known in the woman’s lifetime but not the underlying cause of death on the death certificate		
	Diabetes	Dementia	Cancer
Ischaemic heart disease	rank = 1, *n* = 384 (21.52%)	rank = 1, *n* = 489 (21.57%)	rank = 1, *n* = 266 (23.48%)
Cancer	rank = 2, *n* = 363 (20.35%)	rank = 4, *n* = 291 (12.84%)	
Other circulatory diseases	rank = 3, *n* = 247 (13.85%)	rank = 3, *n* = 372 (16.41%)	rank = 2, *n* = 206 (18.18%)
Dementia	rank = 4, *n* = 194 (10.87%)		rank = 4, *n* = 140 (12.36%)
Cerebrovascular disease	rank = 5, *n* = 192 (10.76%)	rank = 2, *n* = 427 (18.84%)	rank = 3, *n* = 177 (15.62%)
Diabetes		rank = 5, *n* = 108 (4.76%)	
Chronic lower respiratory disease			rank = 5, *n* = 62 (5.47%)

### Sensitivity analysis results

Women with a record of diabetes in their lifetime who were identified from different data sources largely overlapped with each other (Table S3), and so did dementia cases (Table S4). The sensitivity, specificity, and positive and negative predictive values of death certifications of diabetes and dementia were robust to the data sources used (see Tables S3 and S4 in Supplementary Material).

## Discussion

Four notable findings emerged in the present study: (1) the sensitivity for diabetes, dementia, and cancer being recorded as the underlying cause of death was poor but improved substantially if contributing causes of death were taken into account; (2) women with dementia who died before 2006 were less likely to have dementia recorded on their death certificates, compared with those who died during 2006–2011 or 2012–2017. This pattern was the opposite for diabetes and cancer; (3) Sociodemographic characteristics were weakly and inconsistently associated with the sensitivity of these conditions recorded on death certificates; and (4) Diabetes and dementia were under reported particularly on the death certificates of women who died from circulatory diseases or cancer.

In this study, we found that diabetes was recorded as the underlying cause of death for only 12.3% of women with diabetes in their lifetime. This was broadly consistent with the sensitivity observed in an US study which enrolled 11 927 diabetics (10%) [5] and another US study which used data from the National Mortality Follow-back Survey (NMFS) (10%) [12], but higher than the sensitivity found in the US Rancho Bernardo Study (6.2%) [4]. The Rancho Bernardo Study, which included 3 209 participants, found that the sensitivity of death certification of diabetes was higher in women than it was in men, which might explain why the authors found a lower sensitivity than we did as our study only had women participants. Whittall et al. investigated the accuracy of death certification of diabetes in 1 084 Caucasian subjects with diabetes who participated in surveys in rural areas in Western Australia from 1978 to 1982, and found 28% of these women had diabetes recorded as the underlying cause of death [13], which was much higher than our result observed in participants from Western Australia (13.4%). Whether this discrepancy indicates a decreasing trend in the accuracy of death certification of diabetes over the past decades in Western Australia warrants future investigation.

The accuracy of death certification of dementia has been evaluated in previous studies [6–8, 14–20]. We used survey data from a well-designed nationally representative cohort study linked to multiple sources of routinely collected healthcare data [21]. In our study, 52.3% of women with dementia in their lifetime had dementia recorded as the underlying or contributing cause of death, which was similar to the sensitivity observed in cohort studies in South London (53.6%) [7] and Finland (44.4%, 95% CI: 13.7, 78.8) [20], but higher than the sensitivity found in other cohort studies in England and Wales (21.0% in the Cognitive Function and Ageing Study I (CFAS I)) [6], and the US (28.4% and 23.8%) [14, 17]. The inconsistency between our finding and the findings from these studies could be due to different time periods studied. In the CFAS studies, the sensitivity of dementia death certification increased from 21.0% in participants recruited in 1989 (CFAS I) to 45.2% in participants recruited in 2008 (CFAS II) [6]. The inconsistency between the finding of our study and the findings of the US studies could also be explained by the different populations studied or different gold standards used. The sensitivity of dementia death certification in US studies was generally found to be low, even in people with end-stage dementia [16].

As the age range of the participants in our study was narrow (i.e., five years), the difference in the sensitivity of death certification of dementia across the three time periods (i.e., < 2006, 2006–2011, and 2012–2017) may not necessarily mean that there was a temporal change in the sensitivity of death certification, but could mean that women with dementia who died at an older age were more likely to have dementia recorded as a cause of death. Similarly, the difference in the sensitivity of death certification of cancer across the three time periods could either be because the sensitivity of death certification of cancer decreased over time or because some women who lived longer had recovered from cancer and died from some other cause. Changes to the automated coding of multiple causes of death occurred in 2006 and 2013. The first of these changes probably explains the changes in sensitivity of dementia coding observed before and after 2006 (see explanatory note #77 of the Australian Bureau of Statistics publication ‘Causes of Death, Australia, 2015’ [22]). However, the 2013 and any subsequent changes in processing, and slight differences between States and Territories, are less likely to explain the temporal changes in sensitivity of recording of causes of death.

Despite diabetes being one of the most important risk factors for cardiovascular diseases, only 40.9% of women with diabetes had diabetes recorded as a cause of death in our study. We observed that diabetes was under reported particularly on the death certificates of women who died from ischaemic heart disease. For these women it is unlikely that diabetes was not related to the ischaemic heart disease and not a contributing cause of death [13]. We also found that diabetes was under reported in women who died from cancer. This finding was in line with the finding of a previous US study conducted among 11 927 people with diabetes [5]. Diabetes has been increasingly recognized as a predictor of deaths from cancer of pancreas, breast, liver, and colon [23, 24].

The reasons for the underreporting of dementia on death certificates are multifaceted. First, cognitive impairment in people with dementia may restrict them from reporting symptoms and seeking medical help [25], which could explain our finding that unpartnered women were less likely to have dementia recorded on their death certificate than partnered women. Second, the co-existence of more than one health condition (i.e., multimorbidity) is especially common in older people [26], making it hard to identify dementia as the single underlying cause of death, especially when dementia co-exists with cardiovascular diseases. Third, having dementia on a death certificate could be used to challenge the validity of someone’s will [27], which may make the certifying doctor less likely to record dementia as a cause of death.

It should be noted that certifier type (i.e., a medical practitioner or a coroner) also affects how multiple cause of death are recorded on death certificates. In Australia, deaths from natural causes (e.g., cancer or diabetes) are usually certified by a medical practitioner, and most deaths caused by unknown or external causes (e.g., suicides) are certified by a coroner. Approximately 86% to 89% of deaths are certified by a medical practitioner [28], and multiple causes of death are more likely to be recorded for deaths certified by a medical practitioner. So multiple causes are more likely to be recorded for deaths involving diabetes, dementia and cancer than external causes. Previous analysis has shown that this cohort was more socio-economically advantaged and had higher relative survival than other women in Australia born in the same period [29]. However, the results shown in Figs. 1, 2 and 3 do not suggest that socio-economic differences affected the sensitivity of recording of causes of death.

The under reporting of conditions on the death certificates (e.g., diabetes, dementia, or cancer) does not only result in the under estimation of mortality burden associated with these diseases, but also could change the underlying cause of death selected based on coding rules in the automated processing. Several recommendations have been proposed to improve the accuracy of death certification. First, as the current format of death certificates may have limited space, and it has been suggested that adding a series of check boxes for the most common conditions to death certificates could improve the underreporting of some conditions (e.g., diabetes) [30]. In Australia electronic formats of death certificate are being adopted in some States/Territories and this allows for more conditions to be mentioned in Part II. Second, more educational support to help medical practitioners to improve the accuracy of death certificates could be helpful. Our results emphasise the importance of considering how causes of death (including underlying and contributing causes of deaths) are recorded, a challenge highlighted by the need to distinguish between death *from* COVID and deaths *with* COVID. Additionally, national statistics reporting both underlying and contributing causes of death would better represent the burden of disease.

Three main limitations of this study should be acknowledged. First, as this study was from ALSWH, only women were included, restricting our capacity to generalize the findings to men. Second, the narrow age range of the participants restricted the generalization of the research findings to all women in Australia. Third, the place of death (e.g., hospital or aged care facility) has been found to be associated with the accuracy of death certification for dementia [6, 7], but we were unable to obtain this information.

## Conclusion

Death rates due to common conditions that occur in clusters of multimorbidity in the elderly are underestimated in national mortality statistics, but would be improved if the multiple causes of death listed on a death certificate were taken into account in the statistics.

## Supplementary Information

**Additional file 1.**

## Data Availability

The data that support the findings of this study are available from ALSWH Data Access Committee but restrictions apply to the availability of these data, which were used under license for the current study, and so are not publicly available. Data are however available from the authors upon reasonable request and with permission of ALSWH Data Access Committee.
