# Supplementary material for: Accuracy of death certifications of diabetes, dementia and cancer in Australia: a population-based cohort study
Source: BMC Public Health. 2022 May 6;22:902. doi: 10.1186/s12889-022-13304-8 (PMC9074356; doi:10.1186/s12889-022-13304-8)
Supplement: Supplementary file 1 — Additional file 1. [file 12889_2022_13304_MOESM1_ESM.docx]

**Supplementary Material**

**Accuracy of death certifications of diabetes, dementia and cancer in Australia: a population-based cohort study**

**Contents**

**Table S1.1** Ascertainment of diabetes in women’s lifetime from multiple sources………………………. 2

**Table S1.2** Ascertainment of dementia in women’s lifetime from multiple sources……………………… 5

**Table S1.3** Ascertainment of cancer in women’s lifetime from multiple sources………………………… 6

**Table S2.** Twenty-two groups of causes of death…………………………………………………………. 7

**Table S3.** Sensitivity analysis results for diabetes………………………………………………………… 8

**Table S4.** Sensitivity analysis results for dementia……………………….................................................. 9

**Table S1.1** Ascertainment of diabetes in women’s lifetime from multiple sources

| Data source | Eligibility criteria | Survey questions or linked data codes |
| --- | --- | --- |
| ALSWH surveys | Reported twice or more  **OR**  Once or more in MBS, PBS, hospital, or aged care | Survey 1 (1996): Have you ever been told by a doctor that you have diabetes (high blood sugar)?  Survey 2 (1999): In the last 3 years have you been diagnosed with or treated for diabetes (high blood sugar)?  Survey 3 (2002): In the last 3 years have you been diagnosed with or treated for diabetes (high blood sugar)?  Survey 4 (2005): In the last 3 years have you been diagnosed with or treated for diabetes (high blood sugar)?  Survey 5 (2008): In the last 3 years have you been diagnosed with or treated for diabetes (high blood sugar)?  Survey 6 (2011): In the last 3 years have you been diagnosed with or treated for diabetes (high blood sugar)? |
| MBS data | Reported one Diabetes Annual Cycle of Care (DACC)  **OR**  Three or more Hb1Ac, eye exam for diabetes, allied health for diabetes | Code Year Details  00259 2018 Professional attendance at consulting rooms patient with established diabetes mellitus  00260 2018 Professional attendance at other than consulting rooms patient with established diabetes mellitus  00261 2018 Professional attendance at consulting rooms patient with established diabetes mellitus  00262 2018 Professional attendance at other than consulting rooms patient with established diabetes mellitus  00263 2018 Professional attendance at consulting rooms patient with established diabetes mellitus  00264 2018 Professional attendance at other than consulting rooms patient with established diabetes mellitus  02509 2001 Out-of-surgery consultation - Completion of a DACC  02517 2001 Level 'b' Professional attendance - requirements for a full year of care of a patient with established diabetes mellitus  02518 2001 Level 'c' Professional attendance - requirements for a full year of care of a patient with established diabetes mellitus  02521 2002 Surgery consultations - completes the requirements for a full year of care of a patient with established diabetes mellitus  02522 2001 Level 'd' Professional attendance - requirements for a full year of care of a patient with established diabetes mellitus  02525 2002 Surgery consultations - completes the requirements for a full year of care of a patient with established diabetes mellitus  02526 2006 Out-of-surgery consultation - completes the minimum requirements of a DACC  02620 2001 Surgery consultations - completes the requirements for a full year of care of a patient with established diabetes mellitus  02622 2001 Long consultation of more than 25 minutes - completes the requirements for a full year of care of a patient with established diabetes mellitus  02624 2001 Prolonged consultation of more than 45 minutes - completes the requirements for a full year of care of a patient with established diabetes mellitus  02631 2001 Out-of-surgery consultations - completes the requirements for a full year of care of a patient with established diabetes mellitus  02633 2001 Long consultation of more than 25 minutes - completes the requirements for a full year of care of a patient with established diabetes mellitus  02635 2001 Prolonged consultation of more than 45 minutes - completes the requirements for a full year of care of a patient with established diabetes mellitus  10915 2003 Professional attendance of more than 15 minutes duration, being the first in a course of attention involving the examination of the eyes, with the instillation of a mydriatic, of a patient with diabetes mellitus  10951 2005 Diabetes education health service provided to a person by an eligible diabetes educator  12325 2017 Assessment of visual acuity and bilateral retinal photography with a non-mydriatic retinal camera, including analysis and reporting of the images for initial or repeat assessment for presence or absence of diabetic retinopathy  12326 2017 Assessment of visual acuity and bilateral retinal photography with a non-mydriatic retinal camera, including analysis and reporting of the images for initial or repeat assessment for presence or absence of diabetic retinopathy  66319 1996 Quantitation of glycosylated haemoglobin performed in the management of established diabetes  66322 1997 Quantitation of glycosylated haemoglobin performed in the management of pre-existing diabetes where the patient is pregnant  66361 1996 Beta-2-microglobulin, caeruloplasmin, haptoglobins, microalbumin in proven diabetes mellitus  66551 1998 Quantitation of glycosylated haemoglobin performed in the management of established diabetes  66554 1998 Quantitation of glycosylated haemoglobin performed in the management of pre-existing diabetes  66557 1998 Quantitation of fructosamine performed in the management of established diabetes  66560 1998 Microalbumin in proven diabetes mellitus - quantitation in urine  73840 2001 Quantitation of glycosylated haemoglobin performed in the management of established diabetes  73844 2018 Quantitation of urinary microalbumin in the management of established diabetes  81100 2007 Diabetes education health service provided to a person by an eligible diabetes educator  81105 2007 Diabetes education health service provided to a person by an eligible diabetes educator  81110 2007 Exercise physiology health service provided to a person by an eligible exercise physiologist for the purposes of assessing a person's suitability for group services for the management of type 2 diabetes  81115 2007 Exercise physiology health service provided to a person by an eligible exercise physiologist, as a group service for the management of type 2 diabetes  81120 2007 Dietetics health service provided to a person by an eligible dietitian for the purposes of assessing a person's suitability for group services for the management of type 2 diabetes  81125 2007 Dietetics health service provided to a person by an eligible dietitian, as a group service for the management of type 2 diabetes  81305 2015 Diabetes education health service provided to a person who is of Aboriginal or Torres Strait Islander |
| PBS data | Reported two or more scripts within 12 months | A10 Anti-diabetic Therapies |
| Hospital data | Reported once or more | ICD-9 249 Secondary diabetes mellitus  ICD-9 250 Diabetes mellitus  ICD-10 E10 Type 1 diabetes mellitus  ICD-10 E11 Type 2 diabetes mellitus  ICD-10 E13 Other specified diabetes mellitus  ICD-10 E14 Unspecified diabetes mellitus |
| Aged care data | Reported once or more | The Aged Care Assessment Programs (ACAP) or The Aged Care Funding Instrument (ACFI) codes  402 Diabetes mellitus - Type 1 (IDDM)  403 Diabetes mellitus - Type 2 (NIDDM)  404 Diabetes mellitus - other specified/unspecified/unable to be specified |

**Table S1.2** Ascertainment of dementia in women’s lifetime from multiple sources

| Data source | Eligibility criteria | Survey questions or linked data codes |
| --- | --- | --- |
| ALSWH surveys | Reported once or more | Survey 2 (1999): In the last 3 years have you been told by a doctor that you have Alzheimer’s disease or dementia?  Survey 3 (2002): In the last 3 years have you been diagnosed with or treated for Alzheimer’s disease or dementia?  Survey 4 (2005): In the last 3 years have you been diagnosed with or treated for Alzheimer’s disease or dementia?  Survey 5 (2008): In the last 3 years have you been diagnosed with or treated for Alzheimer’s disease or dementia?  Survey 6 (2011): In the last 3 years have you been diagnosed with or treated for Alzheimer’s disease or dementia? |
| PBS data | Reported once or more | N06DA01 Tacrine  N06DA02 Donepezil  N06DA03 Rivastigmine  N06DA04 Galantamine  N06DA52 Donepezil and memantine  N06DA53 Donepezil, memantine and Ginkgo folium  N06DX01 Memantine |
| Hospital data | Reported once or more | ICD-9 290 Dementias  ICD-9 294.2 Dementia, unspecified  ICD-9 331.0 Alzheimer's disease  ICD-10 F00 Dementia in Alzheimer disease  ICD-10 F01 Vascular dementia  ICD-10 F03 Unspecified dementia  ICD-10 G30 Alzheimer's disease |
| Aged care data | Reported once or more | The Aged Care Assessment Programs (ACAP) or The Aged Care Funding Instrument (ACFI) codes  50 Dementia in Alzheimer’s disease  51 Vascular dementia  532 Unspecified dementia |

**Table S1.3** Ascertainment of cancer in women’s lifetime from multiple sources

| Data source | Eligibility criteria | Survey questions or linked data codes |
| --- | --- | --- |
| Cancer Registry data | Reported once | All records in cancer registry |

**Table S2.** Twenty-two groups of causes of death

| **Causes of death** | **ICD-9 codes** | **ICD-10 codes** |
| --- | --- | --- |
| Infectious and parasitic diseases | 001-139 | All A and B codes |
| Cancer | 140-209 | C00-C97 |
| Diabetes | 249, 250 | E10, E11, E13, E14 |
| Other Endocrine, nutritional and metabolic diseases | 240-246, 251-279 | All other E codes |
| Dementia | 290, 294.2, 331.0 | F00, F01, F03, G30 |
| Other mental and behavioural disorders | 290-319 (except for 290 and 294.2) | All other F codes (except F00, F01, F03) |
| Parkinson’s disease | 332 | G20 |
| Other Diseases of the nervous system | 320-389 (except for 331.0 and 332) | All other G codes (except G20 and G30) |
| Ischaemic heart disease | 410-414 | I20-I25 |
| Cerebrovascular disease | 430-438 | I60-I69 |
| Other circulatory diseases | 390-459 (except for 410-414 and 430-438) | All other I codes |
| Pneumonia | 480-486 | J12-J18 |
| Chronic lower respiratory disease | 490-496 | J40-J47 |
| Other respiratory disease | 460-519 (except for 480-486 and 490-496) | All other J codes |
| Liver disease | 570-573 | K70-K77 |
| Other diseases of the digestive system | 520-579 (except for 570-573) | All other K codes |
| Kidney disease | 580-599 | N00-N39 |
| Other Diseases of the genitourinary system | 610-612, 614-616, and 617-629 | All other N codes |
| Falls | E88 | W00-W19 |
| Accidents | E89-E92 | V01-99, W20-W99, X00-X59 |
| Intentional self-harm | E95 | X60-X84 |
| All other causes | Everything else | Everything else |

**Table S3.** Sensitivity analysis results for diabetes

|  | ALSWH surveys | MBS | PBS | Hospital | Aged care | All sources |
| --- | --- | --- | --- | --- | --- | --- |
| Number of diabetes cases from this source | 1 440 | 1 613 | 1 199 | 1 674 | 1 028 | 2 164 |
| Number of diabetes cases exclusively from this source | 44 | 125 | 25 | 177 | 36 | 2 164 |
| Accuracy of death certification of diabetes (diabetes as the underlying cause of death) after leaving this source out |  |  |  |  |  |  |
| *Sensitivity (%)* | 12.3 (10.9, 13.7) | 13.0 (11.5, 14.4) | 12.4 (11.0, 13.8) | 13.2 (11.8, 14.7) | 12.6 (11.1, 14.0) | 12.3 (11.0, 13.7) |
| *Specificity (%)* | 99.9 (99.8, 100.0) | 99.9 (99.9, 100.0) | 99.9 (99.9, 100.0) | 99.9 (99.8, 100.0) | 100.0 (99.9, 100.0) | 100.0 (99.9, 100.0) |
| *Positive predictive value (%)* | 96.7 (94.5, 98.8) | 97.8 (96.0, 99.5) | 98.5 (97.1, 100.0) | 97.4 (95.5, 99.3) | 98.9 (97.6, 100.0) | 98.9 (97.6, 100.0) |
| *Negative predictive value (%)* | 78.8 (78.0, 79.7) | 79.8 (79.0, 80.6) | 78.7 (77.8, 79.5) | 80.4 (79.6, 81.2) | 78.8 (78.0, 79.7) | 78.4 (77.6, 79.3) |
| Accuracy of death certification of diabetes (diabetes as the underlying or contributing cause of death) after leaving this source out |  |  |  |  |  |  |
| *Sensitivity (%)* | 40.1 (38.1, 42.2) | 43.0 (40.9, 45.2) | 41.1 (39.0, 43.2) | 43.4 (41.3, 45.6) | 41.5 (39.4, 43.5) | 40.9 (38.8, 42.9) |
| *Specificity (%)* | 99.2 (99.0, 99.4) | 99.6 (99.4, 99.7) | 99.6 (99.4, 99.7) | 99.4 (99.2, 99.6) | 99.6 (99.5, 99.8) | 99.7 (99.5, 99.8) |
| *Positive predictive value (%)* | 93.7 (92.1, 95.3) | 96.6 (95.4, 97.8) | 96.8 (95.7, 98.0) | 95.0 (93.6, 96.5) | 97.1 (96.1, 98.2) | 97.4 (96.3, 98.4) |
| *Negative predictive value (%)* | 84.4 (83.6, 85.2) | 85.7 (85.0, 86.5) | 84.5 (83.8, 85.3) | 86.2 (85.5, 87.0) | 84.7 (83.9, 85.5) | 84.3 (83.5, 85.1) |

**Table S4.** Sensitivity analysis results for dementia

|  | ALSWH surveys | PBS | Hospital | Aged care | All sources |
| --- | --- | --- | --- | --- | --- |
| Number of dementia cases from this source | 1 064 | 913 | 2 134 | 2 449 | 3 317 |
| Number of dementia cases exclusively from this source | 158 | 48 | 495 | 617 | 3 317 |
| Accuracy of death certification of dementia (dementia as the underlying cause of death) after leaving this source out |  |  |  |  |  |
| *Sensitivity (%)* | 25.6 (24.1, 27.2) | 25.4 (23.9, 26.9) | 27.9 (26.2, 29.5) | 27.1 (25.4, 28.8) | 25.2 (23.7, 26.7) |
| *Specificity (%)* | 98.4 (98.1, 98.7) | 98.7 (98.4, 99.0) | 98.1 (97.8, 98.5) | 97.3 (96.9, 97.7) | 98.8 (98.5, 99.1) |
| *Positive predictive value (%)* | 89.7 (87.7, 91.7) | 91.8 (90.0, 93.6) | 87.2 (85.0, 89.3) | 81.1 (78.5, 83.6) | 92.5 (90.8, 94.2) |
| *Negative predictive value (%)* | 71.2 (70.2, 72.2) | 70.1 (69.1, 71.1) | 75.0 (74.1, 76.0) | 75.9 (74.9, 76.8) | 69.6 (68.6, 70.6) |
| Accuracy of death certification of dementia (dementia as the underlying or contributing cause of death) after leaving this source out |  |  |  |  |  |
| *Sensitivity (%)* | 53.2 (51.4, 54.9) | 52.5 (50.8, 54.2) | 56.1 (54.3, 58.0) | 55.6 (53.7, 57.5) | 52.3 (50.6, 54.0) |
| *Specificity (%)* | 96.2 (95.8, 96.7) | 96.8 (96.4, 97.3) | 94.9 (94.4, 95.5) | 93.7 (93.1, 94.3) | 97.1 (96.6, 97.5) |
| *Positive predictive value (%)* | 88.3 (86.9, 89.8) | 90.3 (88.9, 91.6) | 83.3 (81.7, 85.0) | 79.0 (77.1, 80.8) | 91.2 (89.9, 92.4) |
| *Negative predictive value (%)* | 79.3 (78.4, 80.3) | 78.3 (77.3, 79.3) | 82.7 (81.8, 83.6) | 83.2 (82.4, 84.1) | 77.9 (76.9, 78.8) |
